# Checklist of the family Chironomidae (Diptera) of Finland

**DOI:** 10.3897/zookeys.441.7461

**Published:** 2014-09-19

**Authors:** Lauri Paasivirta

**Affiliations:** 1 Ruuhikoskenkatu 17 B 5, FI-24240 Salo, Finland

**Keywords:** Finland, Chironomidae, species list, biodiversity, faunistics

## Abstract

A checklist of the family Chironomidae (Diptera) recorded from Finland is presented.

## Introduction

There are supposedly at least 15 000 species of chironomid midges in the world (Armitage et al. 1995, but see Pape et al. 2011) making it the largest family among the aquatic insects. The European chironomid fauna consists of 1262 species (Sæther and Spies 2013). In Finland, 780 species can be found, of which 37 are still undescribed (Paasivirta 2012).

The species checklist written by B. Lindeberg on 23.10.1979 (Hackman 1980) included 409 chironomid species. Twenty of those species have been removed from the checklist due to various reasons. The total number of species increased in the 1980s to 570, mainly due to the identification work by me and J. Tuiskunen (Bergman and Jansson 1983, Tuiskunen and Lindeberg 1986). There has been a new rapid increase in the total number of species since 1998. I have reported 187 additional species, of which 8 have been new to science, and other researchers have reported 24 additional species. The chironomid fauna of all biogeographical provinces has been relatively well surveyed (Paasivirta 2012, including the trapping efficiency and the collectors).

Holotypes and paratypes exist for 87 species described from Finland. 23 species have been found only in Finland, thus making them putatively endemic species. Six of these species have only been found from the original type locality. 42 species have been found only from one collecting site in Finland.

Larvae of chironomid midges are found in all types of aquatic habitats. In addition, some species (130 species, 17%) specialize in semi-aquatic environments like wet moss on wetlands and shores of lotic waters. A total of 367 species have been identified from springs and the surrounding habitats in Finland. Sixteen percent of these species are associated with or specialized in springs and 25% are semi-aquatic species (Paasivirta 2007). One hundred and seventy aquatic species have been found from marine habitats, excluding river estuaries (Paasivirta 2000 and later additions). Sixty five additional species live in the mildly brackish water in the northern part of Bothnian Bay. There are only four strictly marine species.

The conservation statuses have been estimated for 667 Finnish chironomid species. Eleven species are classified as vulnerable (VU), 21 as near threatened (NT) and 6 species as lacking sufficient information (DD) (Penttinen et al. 2010). Since the chironomid midges have not been extensively studied and no long-term studies have been performed in Finland, the only useful criteria for the assessment of the conservation statuses have been either extent of occurrence or area of occupancy.

The nomenclature of the species list is consistent with Fauna Europaea (Sæther and Spies 2013). Undescribed species are specified with appellations derived from the first collecting locality. For example *Procladius* sp. 2 “Palsa” was first found at a site called Palsa.

The list of Finnish chironomids (with bioprovincial distribution data) was last updated by Paasivirta (2012).

**Number of species:**

World: 7053 species (Pape et al. 2011)

Europe: 1262 species (Sæther and Spies 2013)

Finland: 780 species, incl. 37 undescribed species

Faunistic knowledge level in Finland: good

## Checklist

Nematocera Dumeril, 1805

infraorder Culicomorpha Hennig, 1948

superfamily Chironomoidea Newman, 1834

**CHIRONOMIDAE** Newman, 1834

PODONOMINAE Thienemann & Edwards, 1937

tribe Boreochlini Brundin, 1966

***BOREOCHLUS*** Edwards, 1938

*Boreochlus thienemanni* Edwards, 1938

***LASIODIAMESA*** Kieffer, 1924

*Lasiodiamesa armata* Brundin, 1966

*Lasiodiamesa gracilis* (Kieffer, 1924)

*Lasiodiamesa sphagnicola* (Kieffer, 1925)

tribe Podonomini Thienemann & Edwards, 1937

***PAROCHLUS*** Enderlein, 1912

*Parochlus kiefferi* (Garrett, 1925)

***TRICHOTANYPUS*** Kieffer, 1906

*Trichotanypus mariae* Wirth & Sublette, 1970

*Trichotanypus posticalis* (Lundbeck, 1898)

TANYPODINAE Skuse, 1889

tribe Anatopyniini Fittkau, 1962

***ANATOPYNIA*** Johannsen, 1905

*Anatopynia plumipes* (Fries, 1823)

tribe Coelotanypodini Coffman, 1978

= Clinotanypodini Lipina, 1928

***CLINOTANYPUS*** Kieffer, 1913

*Clinotanypus nervosus* (Meigen, 1818)

tribe Macropelopiini Zavřel, 1929

***APSECTROTANYPUS*** Fittkau, 1962

*Apsectrotanypus trifascipennis* (Zetterstedt, 1838)

***MACROPELOPIA*** Thienemann, 1916

*Macropelopia adaucta* Kieffer, 1916

= *goetghebueri* (Kieffer, 1918)

*Macropelopia nebulosa* (Meigen, 1804)

*Macropelopia notata* (Meigen, 1818)

***PSECTROTANYPUS*** Kieffer, 1909

*Psectrotanypus varius* (Fabricius, 1787)

tribe Natarsiini Roback & Moss, 1978

***NATARSIA*** Fittkau, 1962

*Natarsia nugax* (Walker, 1856)

*Natarsia punctata* (Fabricius, 1805)

tribe Pentaneurini Hennig, 1950

***ABLABESMYIA*** Johannsen, 1905

*Ablabesmyia longistyla* Fittkau, 1962

*Ablabesmyia monilis* (Linnaeus, 1758)

*Ablabesmyia phatta* (Egger, 1864)

***ARCTOPELOPIA*** Fittkau, 1962

*Arctopelopia barbitarsis* (Zetterstedt, 1850)

*Arctopelopia griseipennis* (van der Wulp, 1859)

*Arctopelopia melanosoma* (Goetghebuer, 1933)

***CONCHAPELOPIA*** Fittkau, 1957

*Conchapelopia aagaardi* Murray, 1987

*Conchapelopia hittmairorum* Michiels & Spies, 2002

*Conchapelopia intermedia* Fittkau, 1962

*Conchapelopia melanops* (Meigen, 1818)

*Conchapelopia pallidula* (Meigen, 1818)

***GUTTIPELOPIA*** Fittkau, 1962

*Guttipelopia guttipennis* (van der Wulp, 1861)

***HAYESOMYIA*** Murray & Fittkau, 1986

*Hayesomyia tripunctata* (Goetghebuer, 1922)

***KRENOPELOPIA*** Fittkau, 1962

*Krenopelopia binotata* (Wiedemann, 1817)

*Krenopelopia nigropunctata* (Staeger, 1839)

***LABRUNDINIA*** Fittkau, 1962

*Labrundinia longipalpis* (Goetghebuer, 1921)

***LARSIA*** Fittkau, 1962

*Larsia atrocincta* (Goetghebuer, 1942)

***MONOPELOPIA*** Fittkau, 1962

*Monopelopia tenuicalcar* (Kieffer, 1918)

***NILOTANYPUS*** Kieffer, 1923

*Nilotanypus dubius* (Meigen, 1804)

***PARAMERINA*** Fittkau, 1962

*Paramerina cingulata* (Walker, 1856)

*Paramerina divisa* (Walker, 1856)

***PENTANEURELLA*** Fittkau & Murray, 1983

*Pentaneurella katterjokki* Fittkau & Murray, 1983

***RHEOPELOPIA*** Fittkau, 1962

*Rheopelopia maculipennis* (Zetterstedt, 1838)

*Rheopelopia ornata* (Meigen, 1838)

***TELMATOPELOPIA*** Fittkau, 1962

*Telmatopelopia nemorum* (Goetghebuer, 1921)

***TELOPELOPIA*** Roback, 1971

*Telopelopia fascigera* (Verneaux, 1970)

***THIENEMANNIMYIA*** Fittkau, 1957

*Thienemannimyia carnea* (Fabricius, 1805)

*Thienemannimyia fusciceps* (Edwards, 1929)

*Thienemannimyia laeta* (Meigen, 1818)

*Thienemannimyia lentiginosa* (Fries, 1823)

*Thienemannimyia pseudocarnea* Murray, 1976

*Thienemannimyia vitellina* (Kieffer, 1916)

***TRISSOPELOPIA*** Kieffer, 1923

*Trissopelopia longimana* (Staeger, 1839)

***XENOPELOPIA*** Fittkau, 1962

*Xenopelopia falcigera* (Kieffer, 1911)

*Xenopelopia nigricans* (Goetghebuer, 1927)

***ZAVRELIMYIA*** Fittkau, 1962

*Zavrelimyia barbatipes* (Kieffer, 1911)

*Zavrelimyia hirtimana* (Kieffer, 1918)

*Zavrelimyia melanura* (Meigen, 1804)

tribe Procladiini Roback, 1971

***PROCLADIUS*** Skuse, 1889

**sg. *Holotanypus*** Roback, 1982

*Procladius appropinquatus* (Lundström, 1917)

*Procladius choreus* (Meigen, 1804)

*Procladius culiciformis* (Linnaeus, 1767)

= *crassinervis* (Zetterstedt, 1838)

*Procladius fimbriatus* Wülker, 1959

*Procladius fuscus* Brundin, 1949

? *Procladius islandicus* (Goetghebuer, 1931)

*Procladius nudipennis* Brundin, 1947

*Procladius pectinatus* (Kieffer, 1909)

*Procladius signatus* (Zetterstedt, 1850)

*Procladius simplicistylus* Freeman, 1948

*Procladius suecicus* Brundin, 1949

Procladius cf. vesus Roback, 1971

*Procladius* sp. 1 “Valassaaret”

*Procladius* sp. 2 “Palsa”

*Procladius* sp. 3 “Kaldoaivi”

**sg. *Psilotanypus*** Kieffer, 1906

*Procladius flavifrons* Edwards, 1929

*Procladius imicola* Kieffer, 1922

*Procladius lugens* Kieffer, 1915

*Procladius rufovittatus* (van der Wulp, 1874)

tribe Tanypodini Skuse, 1889

***TANYPUS*** Meigen, 1803

*Tanypus kraatzi* (Kieffer, 1912)

*Tanypus punctipennis* Meigen, 1818

*Tanypus vilipennis* (Kieffer, 1918)

DIAMESINAE Kieffer, 1922

tribe Diamesini Kieffer, 1922

***DIAMESA*** Meigen, 1835

*Diamesa aberrata* Lundbeck, 1898

*Diamesa arctica* (Boheman, 1865)

*Diamesa bertrami* Edwards, 1935

*Diamesa bohemani* Goetghebuer, 1932

*Diamesa hyperborea* Holmgren, 1869

*Diamesa incallida* (Walker, 1856)

*Diamesa insignipes* Kieffer, 1908

*Diamesa latitarsis* (Goetghebuer, 1921)

*Diamesa permacra* (Walker, 1856)

*Diamesa serratosioi* Willassen, 1986

*Diamesa tonsa* (Haliday, 1856)

= *thienemanni* Kieffer, 1909

***POTTHASTIA*** Kieffer, 1922

*Potthastia gaedii* (Meigen, 1838)

*Potthastia longimanus* Kieffer, 1922

*Potthastia pastoris* (Edwards, 1933)

***PSEUDODIAMESA*** Goetghebuer, 1939

*Pseudodiamesa branickii* (Nowicki, 1873)

*Pseudodiamesa nivosa* (Goetghebuer, 1928)

***PSEUDOKIEFFERIELLA*** Zavřel, 1941

= ***Diplomesa*** Zavřel 1941

*Pseudokiefferiella parva* (Edwards, 1932)

***SYMPOTTHASTIA*** Pagast, 1947

*Sympotthastia fulva* (Johannsen, 1921)

*Sympotthastia huldeni* Tuiskunen, 1986

***SYNDIAMESA*** Kieffer, 1918

*Syndiamesa* sp., indet. larva

tribe Protanypini Brundin, 1956

***PROTANYPUS*** Kieffer, 1906

*Protanypus caudatus* Edwards, 1924

*Protanypus morio* (Zetterstedt, 1838)

TELMATOGETONINAE Wirth, 1949

***TELMATOGETON*** Schiner, 1866

*Telmatogeton japonicus* Tokunaga, 1933

PRODIAMESINAE Sæther, 1976

***MONODIAMESA*** Kieffer, 1922

*Monodiamesa bathyphila* (Kieffer, 1918)

*Monodiamesa ekmani* (Brundin, 1949)

***ODONTOMESA*** Pagast, 1947

*Odontomesa fulva* (Kieffer, 1919)

***PRODIAMESA*** Kieffer, 1906

*Prodiamesa olivacea* (Meigen, 1818)

ORTHOCLADIINAE Kieffer, 1911

***AAGAARDIA*** Sæther, 2000

*Aagaardia protensa* Sæther, 2000

*Aagaardia sivertseni* (Aagaard, 1979)

*Aagaardia* (?) sp. 1

***ABISKOMYIA*** Edwards, 1937

*Abiskomyia paravirgo* Goetghebuer, 1940

*Abiskomyia virgo* Edwards, 1937

***ACAMPTOCLADIUS*** Brundin, 1956

*Acamptocladius reissi* Cranston & Sæther, 1982

*Acamptocladius submontanus* (Edwards, 1932)

***ACRICOTOPUS*** Kieffer, 1921

*Acricotopus lucens* (Zetterstedt, 1850)

***ALLOCLADIUS*** Kieffer, 1913

*Allocladius bothnicus* (Tuiskunen, 1984)

***BOREOSMITTIA*** Tuiskunen, 1986

*Boreosmittia inariensis* Tuiskunen, 1986

*Boreosmittia karelioborealis* Tuiskunen, 1986

***BRILLIA*** Kieffer, 1913

*Brillia bifida* (Kieffer, 1909)

= *modesta* (Meigen, 1830) preocc.

*Brillia longifurca* Kieffer, 1921

***BRYOPHAENOCLADIUS*** Thienemann, 1934

*Bryophaenocladius aestivus* (Brundin, 1947)

*Bryophaenocladius dentatus* (Karl, 1937)

*Bryophaenocladius flexidens* (Brundin, 1947)

*Bryophaenocladius ictericus* (Meigen, 1830)

*Bryophaenocladius illimbatus* (Edwards, 1929)

Bryophaenocladius cf. impectinus Sæther, 1976

*Bryophaenocladius inconstans* (Brundin, 1947)

Bryophaenocladius cf. laticaudus Sæther, 1973

*Bryophaenocladius muscicola* (Kieffer, 1906)

*Bryophaenocladius nidorum* (Edwards, 1929)

*Bryophaenocladius nigrus* Albu, 1974

*Bryophaenocladius nitidicollis* (Goetghebuer, 1913)

*Bryophaenocladius pectinatus* Albu, 1974

*Bryophaenocladius psilacrus* Sæther, 1982

*Bryophaenocladius saanae* Tuiskunen, 1986

*Bryophaenocladius scanicus* (Brundin, 1947)

Bryophaenocladius cf. sclerus Wang, Liu & Epler, 2004

*Bryophaenocladius subparallelus* (Malloch, 1915)

*Bryophaenocladius subvernalis* (Edwards, 1929)

*Bryophaenocladius tuberculatus* (Edwards, 1929)

*Bryophaenocladius vernalis* (Goetghebuer, 1921)

*Bryophaenocladius xanthogyne* (Edwards, 1929)

Bryophaenocladius sp., pr. xanthogyne

*Bryophaenocladius* sp. 1 ”Syöte”

*Bryophaenocladius* sp. 2 ”Syöte”

*Bryophaenocladius* sp. 3 ”Malla”

*Bryophaenocladius* sp. 4 ”Tsarmi”

*Bryophaenocladius* sp. 5 ”Pallas”

*Bryophaenocladius* sp. 6 ”Rommas”

***CAMPTOCLADIUS*** van der Wulp, 1874

*Camptocladius stercorarius* (De Geer, 1776)

***CARDIOCLADIUS*** Kieffer, 1912

*Cardiocladius capucinus* (Zetterstedt, 1850)

*Cardiocladius fuscus* Kieffer, 1924

***CHAETOCLADIUS*** Kieffer, 1911

*Chaetocladius acuminatus* Brundin, 1956

*Chaetocladius binotatus* (Lundström, 1915)

*Chaetocladius britae* Säwedal, 1976

*Chaetocladius crassisaetosus* Tuiskunen, 1986

*Chaetocladius dentiforceps* (Edwards, 1929)

*Chaetocladius dissipatus* (Edwards, 1929)

*Chaetocladius glacialis* (Lundström, 1915)

*Chaetocladius gracilis* Brundin, 1956

*Chaetocladius grandilobus* Brundin, 1956

*Chaetocladius laminatus* Brundin, 1947

*Chaetocladius maeaeri* Brundin, 1947

Chaetocladius sp., pr. melaleucus (Meigen, 1818)

*Chaetocladius muliebris* Tuiskunen, 1986

*Chaetocladius perennis* (Meigen, 1830)

*Chaetocladius piger* (Goetghebuer, 1913)

Chaetocladius cf. rusticus (Goetghebuer, 1932)

*Chaetocladius suecicus* (Kieffer, 1916)

*Chaetocladius tenuistylus* Brundin, 1947

***CLUNIO*** Haliday, 1855

*Clunio balticus* Heimbach, 1978

***CORYNONEURA*** Winnertz, 1846

*Corynoneura arctica* Kieffer, 1923

*Corynoneura brundini* Hirvenoja & Hirvenoja, 1988

*Corynoneura carriana* Edwards, 1924

*Corynoneura celeripes* Winnertz, 1852

*Corynoneura celtica* Edwards, 1924

*Corynoneura coronata* Edwards, 1924

*Corynoneura edwardsi* Brundin, 1949

*Corynoneura fittkaui* Schlee, 1968

*Corynoneura gratias* Schlee, 1968

*Corynoneura gynocera* Tuiskunen, 1983

*Corynoneura lacustris* Edwards, 1924

*Corynoneura lobata* Edwards, 1924

*Corynoneura magna* Brundin, 1949

*Corynoneura scutellata* Winnertz, 1846

= *longipennis* auct. nec Tokunaga, 1936

*Corynoneura* sp. 1 “Tvärminne”

*Corynoneura* sp. 2

***CORYNONEURELLA*** Brundin, 1949

*Corynoneurella paludosa* Brundin, 1949

***CRICOTOPUS*** van der Wulp, 1874

**sg. *Cricotopus*** van der Wulp, 1874

*Cricotopus albiforceps* (Kieffer, 1916)

*Cricotopus annulator* Goetghebuer, 1927

*Cricotopus bicinctus* (Meigen, 1818)

*Cricotopus caducus* Hirvenoja, 1973

*Cricotopus coronatus* Hirvenoja, 1973

*Cricotopus cumulatus* Hirvenoja, 1973

*Cricotopus cylindraceus* (Kieffer, 1908)

*Cricotopus ephippium* (Zetterstedt, 1838)

*Cricotopus festivellus* (Kieffer, 1906)

*Cricotopus flavocinctus* (Kieffer, 1924)

*Cricotopus fuscus* (Kieffer, 1909)

*Cricotopus magus* Hirvenoja, 1973

*Cricotopus pallidipes* Edwards, 1929

*Cricotopus patens* Hirvenoja, 1973

*Cricotopus pilidorsum* Hirvenoja, 1973

*Cricotopus pilosellus* Brundin, 1956

*Cricotopus pirifer* Hirvenoja, 1973

*Cricotopus polaris* Kieffer, 1926

Cricotopus sp., pr. polaris

*Cricotopus pulchripes* Verrall, 1912

*Cricotopus septentrionalis* Hirvenoja, 1973

*Cricotopus similis* Goetghebuer, 1921

*Cricotopus slossonae* Malloch, 1915

= *claripes* Hirvenoja, 1973

*Cricotopus tibialis* (Meigen, 1804)

*Cricotopus tremulus* (Linnaeus, 1758)

*Cricotopus triannulatus* (Macquart, 1826)

*Cricotopus trifascia* Edwards, 1929

*Cricotopus tristis* Hirvenoja, 1973

*Cricotopus vierriensis* Goetghebuer, 1935

*Cricotopus villosus* Hirvenoja, 1973

**sg. *Isocladius*** Kieffer, 1909

*Cricotopus arcuatus* Hirvenoja, 1973

*Cricotopus brevipalpis* Kieffer, 1909

*Cricotopus intersectus* (Staeger, 1839)

*Cricotopus laetus* Hirvenoja, 1973

*Cricotopus laricomalis* Edwards, 1932

*Cricotopus maurii* Spies & Sæther, 2004

= *polychaetus* Hirvenoja, 1989 preocc.

*Cricotopus obnixus* (Walker, 1856)

Cricotopus sp. cf. obnixus Hirvenoja, 1973

*Cricotopus obtusus* Hirvenoja, 1973

*Cricotopus ornatus* (Meigen, 1818)

*Cricotopus perniger* (Zetterstedt, 1850)

*Cricotopus pilicauda* Hirvenoja, 1973

*Cricotopus pilitarsis* (Zetterstedt, 1850)

*Cricotopus reductus* Hirvenoja, 1973

*Cricotopus relucens* Hirvenoja, 1973

*Cricotopus reversus* Hirvenoja, 1973

*Cricotopus suspiciosus* Hirvenoja, 1973

*Cricotopus sylvestris* (Fabricius, 1794)

*Cricotopus tricinctus* (Meigen, 1818)

*Cricotopus trifasciatus* (Meigen, 1810)

**sg. *Nostococladius*** Ashe & Murray, 1980

*Cricotopus lygropis* Edwards, 1929

**sg. ?**

*Cricotopus* (?) sp. 1 “Tapionaho”

***DIPLOCLADIUS*** Kieffer, 1908

*Diplocladius cultriger* Kieffer, 1908

***DONCRICOTOPUS*** Sæther, 1981

*Doncricotopus dentatus* Tuiskunen, 1985

***DRATNALIA*** Sæther & Halvorsen, 1981

*Dratnalia potamophylaxi* (Fittkau & Lellak, 1971)

***EPOICOCLADIUS*** Sulc & Zavřel, 1924

*Epoicocladius ephemerae* (Kieffer, 1924)

= *flavens* auct. nec (Malloch, 1915)

***EUKIEFFERIELLA*** Thienemann, 1926

*Eukiefferiella boevrensis* Brundin, 1956

*Eukiefferiella brevicalcar* (Kieffer, 1911)

*Eukiefferiella claripennis* (Lundbeck, 1898)

*Eukiefferiella clypeata* (Thienemann, 1919)

*Eukiefferiella devonica* (Edwards, 1929)

*Eukiefferiella dittmari* Lehmann, 1972

*Eukiefferiella gracei* (Edwards, 1929)

Eukiefferiella sp., pr. gracei

*Eukiefferiella ilkleyensis* (Edwards, 1929)

*Eukiefferiella minor* (Edwards, 1929)

***EURYCNEMUS*** van der Wulp, 1874

*Eurycnemus crassipes* (Meigen, 1810)

***GEORTHOCLADIUS*** Strenzke, 1941

*Georthocladius luteicornis* (Goetghebuer, 1941)

*Georthocladius platystylus* Sæther & Sublette, 1983

?= *Parachaetocladius retezati* Albu, 1972

***GYMNOMETRIOCNEMUS*** Edwards, 1932

**sg. *Gymnometriocnemus*** Edwards, 1932

*Gymnometriocnemus subnudus* (Edwards, 1929)

**sg. *Rhaphidocladius*** Sæther, 1983

*Gymnometriocnemus brumalis* (Edwards, 1929)

*Gymnometriocnemus volitans* (Goetghebuer, 1940)

***HALOCLADIUS*** Hirvenoja, 1973

*Halocladius variabilis* (Staeger, 1839)

***HELENIELLA*** Gowin, 1943

*Heleniella ornaticollis* (Edwards, 1929)

*Heleniella serratosioi* Ringe, 1976

***HETEROTANYTARSUS*** Spärck, 1923

*Heterotanytarsus apicalis* (Kieffer, 1921)

*Heterotanytarsus brundini* Fittkau, 1956

*Heterotanytarsus* sp. 1

***HETEROTRISSOCLADIUS*** Spärck, 1923

*Heterotrissocladius brundini* Sæther & Schnell, 1988

*Heterotrissocladius grimshawi* (Edwards, 1929)

*Heterotrissocladius maeaeri* Brundin, 1949

*Heterotrissocladius marcidus* (Walker, 1856)

*Heterotrissocladius subpilosus* (Kieffer, 1911)

*Heterotrissocladius* sp. 1 ”Kolmperä”

***HYDROBAENUS*** Fries, 1830

*Hydrobaenus conformis* (Holmgren, 1869)

*Hydrobaenus fusistylus* (Goetghebuer, 1933)

*Hydrobaenus lapponicus* (Brundin, 1956)

*Hydrobaenus martini* Sæther, 1976

*Hydrobaenus pilipes* (Malloch, 1915)

*Hydrobaenus spinnatis* Sæther, 1976

*Hydrobaenus* (?) sp. 1

***HYDROSMITTIA*** Ferrington & Sæther, 2011

*Hydrosmittia oxoniana* (Edwards, 1922)

= *recta* (Edwards, 1929)

*Hydrosmittia ruttneri* (Strenzke & Thienemann, 1942)

= *brevitarsis* (Brundin, 1947)

***KRENOSMITTIA*** Thienemann & Krüger, 1939

*Krenosmittia boreoalpina* (Goetghebuer, 1944)

*Krenosmittia camptophleps* (Edwards, 1929)

*Krenosmittia halvorseni* (Cranston & Sæther, 1986)

***LAPPOKIEFFERIELLA*** Tuiskunen, 1986

*Lappokiefferiella platytarsus* Tuiskunen, 1986

***LIMNOPHYES*** Eaton, 1875

*Limnophyes aagaardi* Sæther, 1990

*Limnophyes angelicae* Sæther, 1990

*Limnophyes asquamatus* Soegaard Andersen, 1937

= *smolandicus* Brundin, 1947

*Limnophyes bidumus* Sæther, 1990

Limnophyes sp., pr. bidumus

*Limnophyes brachytomus* (Kieffer, 1922)

*Limnophyes difficilis* Brundin, 1947

*Limnophyes edwardsi* Sæther, 1990

*Limnophyes er* Sæther, 1985

*Limnophyes habilis* (Walker, 1856)

= *truncorum* Goetghebuer, 1921

*Limnophyes margaretae* Sæther, 1975

*Limnophyes minimus* (Meigen, 1818)

= *exiguus* (Goetghebuer, 1913)

*Limnophyes natalensis* (Kieffer, 1914)

*Limnophyes ninae* Sæther, 1975

*Limnophyes pentaplastus* (Kieffer, 1921)

*Limnophyes pumilio* (Holmgren, 1869)

= *globifer* Lundström, 1915

Limnophyes sp., pr. pumilio

*Limnophyes schnelli* Sæther, 1990

*Limnophyes spinigus* Sæther, 1990

*Limnophyes torulus* Sæther, 1990

***MESOCRICOTOPUS*** Brundin, 1956

*Mesocricotopus thienemanni* (Goetghebuer, 1940)

***MESOSMITTIA*** Brundin, 1956

*Mesosmittia flexuella* (Edwards, 1929)

***METRIOCNEMUS*** van der Wulp, 1874

*Metriocnemus acutus* Sæther, 1995

*Metriocnemus albolineatus* (Meigen, 1818)

= *atratulus* (Zetterstedt, 1850)

*Metriocnemus atriclava* Kieffer, 1921

*Metriocnemus beringensis* (Cranston & Oliver, 1988)

*Metriocnemus caudigus* Sæther, 1995

*Metriocnemus corticalis* Strenzke, 1950

*Metriocnemus eurynotus* (Holmgren, 1883)

= *hygropetricus* Kieffer, 1912

*Metriocnemus exilacies* Sæther, 1995

*Metriocnemus fuscipes* (Meigen, 1818)

*Metriocnemus intergerivus* Sæther, 1995

*Metriocnemus picipes* (Meigen, 1818)

*Metriocnemus ursinus* (Holmgren, 1869)

***NANOCLADIUS*** Kieffer, 1913

= ***Microcricotopus*** Thienemann & Harnisch, 1932

*Nanocladius balticus* (Palmén, 1959)

*Nanocladius dichromus* (Kieffer, 1906)

= *bicolor* (Zetterstedt, 1838) nom. nudum

*Nanocladius parvulus* (Kieffer, 1909)

*Nanocladius rectinervis* (Kieffer, 1911)

***ORTHOCLADIUS*** van der Wulp, 1874

**sg. *Eudactylocladius*** Thienemann, 1935

*Orthocladius fuscimanus* (Kieffer, 1908)

*Orthocladius gelidorum* (Kieffer, 1923)

*Orthocladius gelidus* Kieffer, 1922

*Orthocladius musester* Sæther, 2004

*Orthocladius olivaceus* (Kieffer, 1911)

= *mixtus* auct., partim

*Orthocladius priomixtus* Sæther, 2004

= *mixtus* auct., partim

**sg. *Euorthocladius*** Thienemann, 1935

*Orthocladius abiskoensis* Thienemann & Krüger, 1937

Orthocladius cf. annellae Sæther, 2005

*Orthocladius ashei* Soponis, 1990

*Orthocladius rivicola* Kieffer, 1911

*Orthocladius rivulorum* Kieffer, 1909

*Orthocladius saxosus* (Tokunaga, 1939)

*Orthocladius telochaetus* Langton, 1985

**sg. *Mesorthocladius*** Sæther, 2005

*Orthocladius frigidus* (Zetterstedt, 1838)

**sg. *Orthocladius*** van der Wulp, 1874

*Orthocladius decoratus* (Holmgren, 1869)

*Orthocladius dentifer* Brundin, 1947

*Orthocladius excavatus* Brundin, 1947

*Orthocladius lapponicus* Goetghebuer, 1940

*Orthocladius nitidoscutellatus* Lundström, 1915

*Orthocladius oblidens* (Walker, 1856)

*Orthocladius pedestris* Kieffer, 1909

*Orthocladius rhyacobius* Kieffer, 1911

*Orthocladius rubicundus* (Meigen, 1818)

= *saxicola* Kieffer, 1911

*Orthocladius wetterensis* Brundin, 1956

**sg. *Pogonocladius*** Brundin, 1956

*Orthocladius consobrinus* (Holmgren, 1869)

**sg. *Symposiocladius*** Cranston, 1982

*Orthocladius holsatus* Goetghebuer, 1937

*Orthocladius lignicola* Kieffer, 1914

*Orthocladius ruffoi* Rossaro & Prato, 1991

*Orthocladius schnelli* Sæther, 2004

*Orthocladius smolandicus* Brundin, 1947

***PARACHAETOCLADIUS*** Wülker, 1959

*Parachaetocladius abnobaeus* (Wülker, 1959)

***PARACLADIUS*** Hirvenoja, 1973

*Paracladius alpicola* (Zetterstedt, 1850)

*Paracladius conversus* (Walker, 1856)

*Paracladius quadrinodosus* Hirvenoja, 1973

***PARACRICOTOPUS*** Brundin, 1956

*Paracricotopus niger* (Kieffer, 1913)

*Paracricotopus uliginosus* (Brundin, 1947)

***PARAKIEFFERIELLA*** Thienemann, 1936

*Parakiefferiella bathophila* (Kieffer, 1912)

*Parakiefferiella bilobata* Tuiskunen, 1986

*Parakiefferiella coronata* (Edwards, 1929)

*Parakiefferiella fennica* Tuiskunen, 1986

*Parakiefferiella finnmarkica* Tuiskunen, 1986

*Parakiefferiella gynocera* (Edwards, 1937)

*Parakiefferiella minuta* Tuiskunen, 1986

*Parakiefferiella nigra* Brundin, 1949

*Parakiefferiella scandica* Brundin, 1956

*Parakiefferiella smolandica* (Brundin, 1947)

*Parakiefferiella subaterrima* (Malloch, 1915)

= *torulata* Sæther, 1969

***PARALIMNOPHYES*** Brundin, 1956

*Paralimnophyes longiseta* (Thienemann, 1919)

= *hydrophilus* (Goetghebuer, 1921)

***PARAMETRIOCNEMUS*** Goetghebuer, 1932

*Parametriocnemus boreoalpinus* Gowin & Thienemann, 1942

*Parametriocnemus stylatus* (Spärck, 1923)

*Parametriocnemus* sp. 1

***PARAPHAENOCLADIUS*** Thienemann, 1924

*Paraphaenocladius exagitans* (Johannsen, 1905)

Paraphaenocladius sp., pr. exagitans

*Paraphaenocladius impensus* (Walker, 1856)

*Paraphaenocladius intercedens* Brundin, 1947

Paraphaenocladius sp., pr. intercedens

*Paraphaenocladius irritus* (Walker, 1856)

*Paraphaenocladius pseudirritus* Strenzke, 1950

*Paraphaenocladius triangulus* Sæther & Wang, 1995

***PARASMITTIA*** Strenzke, 1950

*Parasmittia carinata* Strenzke, 1950

*Parasmittia* sp. 1 ”Luukki”

***PARATRICHOCLADIUS*** Santos Abreu, 1918

*Paratrichocladius rufiventris* (Meigen, 1830)

*Paratrichocladius skirwithensis* (Edwards, 1929)

***PARATRISSOCLADIUS*** Zavřel, 1937

*Paratrissocladius excerptus* (Walker, 1856)

***PROPSILOCERUS*** Kieffer, 1923

= ***Synpsilocerus*** Kieffer, 1923

= ***Tokunagayusurika*** Sasa, 1978

*Propsilocerus jacuticus* (Zvereva, 1950)

*Propsilocerus komensis* (Zvereva, 1950)

?= *paradoxus* (Lundström, 1915)

*Propsilocerus saetheri* Wang, Liu & Paasivirta, 2007

***PROSMITTIA*** Brundin, 1956

*Prosmittia jemtlandica* (Brundin, 1947)

*Prosmittia rectangularis* Tuiskunen, 1985

***PSECTROCLADIUS*** Kieffer, 1906

**sg. *Allopsectrocladius*** Wülker, 1956

*Psectrocladius conjungens* (Brundin, 1947)

*Psectrocladius obvius* (Walker, 1856)

*Psectrocladius platypus* (Edwards, 1929)

**sg. *Mesopsectrocladius*** Laville, 1971

*Psectrocladius barbatipes* Kieffer, 1923

**sg. *Monopsectrocladius*** Wülker, 1956

*Psectrocladius calcaratus* (Edwards, 1929)

**sg. *Psectrocladius*** Kieffer, 1906

*Psectrocladius barbimanus* (Edwards, 1929)

*Psectrocladius bisetus* Goetghebuer, 1942

*Psectrocladius fennicus* Storå, 1939

*Psectrocladius limbatellus* (Holmgren, 1869)

= *edwardsi* Brundin, 1949

*Psectrocladius octomaculatus* Wülker, 1956

*Psectrocladius oligosetus* Wülker, 1956

*Psectrocladius oxyura* Langton, 1985

*Psectrocladius psilopterus* (Kieffer, 1906)

*Psectrocladius schlienzi* Wülker, 1956

*Psectrocladius sordidellus* (Zetterstedt, 1838)

*Psectrocladius ventricosus* Kieffer, 1925

*Psectrocladius zetterstedti* Brundin, 1949

***PSEUDORTHOCLADIUS*** Goetghebuer, 1943

*Pseudorthocladius curtistylus* (Goetghebuer, 1921)

*Pseudorthocladius filiformis* (Kieffer, 1908)

*Pseudorthocladius pilosipennis* Brundin, 1956

***PSEUDOSMITTIA*** Edwards, 1932

*Pseudosmittia albipennis* (Goetghebuer, 1921)

*Pseudosmittia angusta* (Edwards, 1929)

*Pseudosmittia danconai* (Marcuzzi, 1947)

*Pseudosmittia forcipata* (Goetghebuer, 1921)

Pseudosmittia sp., pr. forcipata

*Pseudosmittia gracilis* (Goetghebuer, 1913)

*Pseudosmittia mathildae* Albu, 1968

*Pseudosmittia obtusa* Strenzke, 1960

*Pseudosmittia trilobata* (Edwards, 1929)

***PSILOMETRIOCNEMUS*** Sæther, 1969

*Psilometriocnemus europaeus* Tuiskunen, 1985

***RHEOCRICOTOPUS*** Brundin, 1956

**sg. *Psilocricotopus*** Sæther, 1986

*Rheocricotopus atripes* (Kieffer, 1913)

= *foveatus* (Edwards, 1929)

*Rheocricotopus chalybeatus* (Edwards, 1929)

*Rheocricotopus chapmani* (Edwards, 1935)

*Rheocricotopus glabricollis* (Meigen, 1830)

**sg. *Rheocricotopus*** Brundin, 1956

*Rheocricotopus effusus* (Walker, 1856)

*Rheocricotopus fuscipes* (Kieffer, 1909)

= *dispar* (Goetghebuer, 1913)

*Rheocricotopus reduncus* Sæther & Schnell, 1988

Rheocricotopus sp., pr. unidentatus Sæther & Schnell, 1988

***RHEOSMITTIA*** Brundin, 1986

*Rheosmittia languida* (Brundin, 1956)

*Rheosmittia spinicornis* (Brundin, 1956)

***SMITTIA*** Holmgren, 1869

*Smittia alpilonga* Rossaro & Lencioni, 2000

Smittia cf. amoena Caspers, 1988

*Smittia aterrima* (Meigen, 1818)

Smittia sp., pr. aterrima

*Smittia betuletorum* Edwards, 1941

*Smittia contingens* (Walker, 1856)

*Smittia edwardsi* Goetghebuer, 1932

*Smittia foliacea* (Kieffer, 1921)

*Smittia leucopogon* (Meigen, 1804)

*Smittia nudipennis* (Goetghebuer, 1913)

*Smittia paranudipennis* Brundin, 1947

*Smittia pratorum* (Goetghebuer, 1927)

*Smittia scutellosetosa* Caspers, 1988

*Smittia stercoraria* Rossaro & Lencioni, 2000

*Smittia* sp. 1 s. Tuiskunen

*Smittia* sp., pr. sp. 1

*Smittia* sp. 2 ”Jehkats”

***STACKELBERGINA*** Shilova & Zelentsov, 1978

*Stackelbergina praeclara* Shilova & Zelentsov, 1978

***STILOCLADIUS*** Rossaro, 1979

*Stilocladius intermedius* Wang, 1998

***SYNORTHOCLADIUS*** Thienemann, 1935

*Synorthocladius semivirens* (Kieffer, 1909)

***TAVASTIA*** Tuiskunen, 1985

*Tavastia australis* Tuiskunen, 1985

*Tavastia yggdrasilia* Brodin, Lundström & Paasivirta, 2008

***THIENEMANNIA*** Kieffer, 1909

*Thienemannia gracilis* Kieffer, 1909

*Thienemannia paasivirtai* Tuiskunen, 1986

***THIENEMANNIELLA*** Kieffer, 1911

*Thienemanniella acuticornis* (Kieffer, 1912)

*Thienemanniella clavicornis* (Kieffer, 1911)

*Thienemanniella majuscula* (Edwards, 1924)

*Thienemanniella minuscula* (Brundin, 1949)

*Thienemanniella obscura* Brundin, 1947

*Thienemanniella vittata* (Edwards, 1924)

Thienemanniella sp., pr. vittata

***TOKUNAGAIA*** Sæther, 1973

*Tokunagaia excellens* (Brundin, 1956)

*Tokunagaia parexcellens* Tuiskunen, 1986

*Tokunagaia rectangularis* (Goetghebuer, 1940)

*Tokunagaia scutellata* (Brundin, 1956)

*Tokunagaia tonollii* (Rossaro, 1983)

***TRISSOCLADIUS*** Kieffer, 1908

*Trissocladius brevipalpis* Kieffer, 1908

***TVETENIA*** Kieffer, 1922

*Tvetenia bavarica* (Goetghebuer, 1934)

*Tvetenia calvescens* (Edwards, 1929)

*Tvetenia discoloripes* (Goetghebuer & Thienemann, 1936)

*Tvetenia duodenaria* Kieffer, 1922

= *saanensis* (Wülker, 1959)

*Tvetenia verralli* (Edwards, 1929)

?= *tshernovskii* (Pankratova, 1968 )

***VIVACRICOTOPUS*** Schnell & Sæther, 1988

*Vivacricotopus ablusus* Schnell & Sæther, 1988

***ZALUTSCHIA*** Lipina, 1939

Zalutschia cf. humphriesiae Dowling & Murray, 1980

*Zalutschia mallae* Tuiskunen, 1986

*Zalutschia mucronata* (Brundin, 1949)

*Zalutschia tatrica* (Pagast, 1935)

*Zalutschia tornetraeskensis* (Edwards & Thienemann, 1941)

*Zalutschia zalutschicola* Lipina, 1939

**genus unknown**

Orthocladiinae sp. 1 ”Outajärvi”

Orthocladiinae sp. 2 ”Pallas”

Orthocladiinae sp. 3 ”Kaldoaivi”

CHIRONOMINAE Newman, 1834

tribe Chironomini Newman, 1834

***BENTHALIA*** Lipina, 1939

*Benthalia carbonaria* (Meigen, 1804)

= *dissidens* (Walker, 1856)

***CHIRONOMUS*** Meigen, 1803

**sg. *Chaetolabis*** Townes, 1945

*Chironomus macani* Freeman, 1948

**sg. *Chironomus*** Meigen, 1803

*Chironomus acerbus* Hirvenoja, 1962

*Chironomus acutiventris* Wülker, Ryser & Scholl, 1983

*Chironomus agilis* Shobanov & Dyomin, 1988

*Chironomus annularius* Meigen, 1818

*Chironomus anthracinus* Zetterstedt, 1860

*Chironomus aprilinus* Meigen, 1818

*Chironomus beljaninae* Wülker, 1991

*Chironomus borokensis* Kerkis, Filippova, Shobanov, Gunderina & Kiknadze, 1988

*Chironomus brevidentatus* Hirvenoja & Michailova, 1998

*Chironomus cingulatus* Meigen, 1830

*Chironomus clarus* Hirvenoja, 1962

*Chironomus coaetaneus* Hirvenoja, 1998

*Chironomus dorsalis* Strenzke, 1959

*Chironomus entis* Shobanov, 1989

*Chironomus esai* Wülker, 1997

*Chironomus fraternus* Wülker, 1991

*Chironomus heteropilicornis* Wülker, 1996

? *Chironomus hyperboreus* Staeger, 1845

*Chironomus inermifrons* Goetghebuer, 1921

*Chironomus islandicus* (Kieffer, 1913)

*Chironomus jonmartini* Lindeberg, 1979) coll.

= *neglectus* Lindeberg, 1960 preocc.

*Chironomus longistylus* Goetghebuer, 1921

*Chironomus lugubris* Zetterstedt, 1850

*Chironomus luridus* Strenzke, 1959

*Chironomus melanescens* Keyl, 1961

*Chironomus melanotus* Keyl, 1961

*Chironomus muratensis* Ryser, Scholl & Wülker, 1983

*Chironomus neocorax* Wülker & Butler, 1983

*Chironomus pallidivittatus* Edwards, 1929

*Chironomus piger* Strenzke, 1956

*Chironomus pilicornis* (Fabricius, 1787)

*Chironomus plumosus* (Linnaeus, 1758)

*Chironomus pseudothummi* Strenzke, 1959 coll.

*Chironomus riihimakiensis* Wülker, 1973

*Chironomus riparius* Meigen, 1804

*Chironomus salinarius* Kieffer, 1915

*Chironomus saxatilis* Wülker, Ryser & Scholl, 1981 coll.

*Chironomus sollicitus* Hirvenoja, 1962

*Chironomus sororius* Wülker, 1973

? *Chironomus staegeri* Lundbeck, 1898

*Chironomus tentans* Fabricius, 1805

*Chironomus tenuistylus* Brundin, 1949

**sg. *Lobochironomus*** Ryser, Wülker & Scholl, 1985

*Chironomus dorsalis* Meigen, 1818

= *longipes* Staeger, 1839 as *Einfeldia*

*Chironomus improvidus* Hirvenoja, 1998

*Chironomus mendax* Storå, 1936

*Chironomus storai* Goetghebuer, 1954

= *luctuosus* Storå, 1936 preocc.

***CLADOPELMA*** Kieffer, 1921

*Cladopelma bicarinatum* (Brundin, 1947)

*Cladopelma edwardsi* (Kruseman, 1933)

*Cladopelma goetghebueri* Spies & Sæther, 2004

= *lateralis* (Goetghebuer, 1934) preocc.

*Cladopelma virescens* (Meigen, 1818)

*Cladopelma viridulum* (Linnaeus, 1767)

***CRYPTOCHIRONOMUS*** Kieffer, 1918

*Cryptochironomus albofasciatus* (Staeger, 1839)

*Cryptochironomus denticulatus* (Goetghebuer, 1921)

*Cryptochironomus obreptans* (Walker, 1856)

*Cryptochironomus psittacinus* (Meigen, 1830)

*Cryptochironomus redekei* (Kruseman, 1933)

*Cryptochironomus rostratus* Kieffer, 1921

*Cryptochironomus supplicans* (Meigen, 1830)

*Cryptochironomus ussouriensis* (Goetghebuer, 1933)

= *nigridens* Chernovskij, 1949

***CRYPTOTENDIPES*** Beck & Beck, 1969

*Cryptotendipes darbyi* (Sublette, 1960)

*Cryptotendipes pflugfelderi* Reiss, 1964

*Cryptotendipes pseudotener* (Goetghebuer, 1922)

*Cryptotendipes usmaensis* (Pagast, 1931)

***DEMEIJEREA*** Kruseman, 1933

*Demeijerea rufipes* (Linnaeus, 1761)

***DEMICRYPTOCHIRONOMUS*** Lenz, 1941

**sg. *Demicryptochironomus*** Lenz, 1941

*Demicryptochironomus vulneratus* (Zetterstedt, 1838)

*Demicryptochironomus* sp. Pe 1 Langton, 1991

**sg. *Irmakia*** Reiss, 1988

*Demicryptochironomus neglectus* Reiss, 1988

***DICROTENDIPES*** Kieffer, 1913

= ***Limnochironomus*** Kieffer, 1920

*Dicrotendipes lobiger* (Kieffer, 1921)

*Dicrotendipes nervosus* (Staeger, 1839)

*Dicrotendipes notatus* (Meigen, 1818)

*Dicrotendipes pulsus* (Walker, 1856)

= *objectans* (Walker, 1856)

*Dicrotendipes tritomus* (Kieffer, 1916)

***EINFELDIA*** Kieffer, 1924

*Einfeldia pagana* (Meigen, 1838)

*Einfeldia pectoralis* Kieffer, 1924

***ENDOCHIRONOMUS*** Kieffer, 1918

*Endochironomus albipennis* (Meigen, 1830)

*Endochironomus stackelbergi* Goetghebuer, 1935

*Endochironomus tendens* (Fabricius, 1775)

***GLYPTOTENDIPES*** Kieffer, 1913

**sg. *Caulochironomus*** Heyn, 1993

*Glyptotendipes aequalis* (Kieffer, 1922)

*Glyptotendipes caulicola* (Kieffer, 1913)

*Glyptotendipes imbecilis* (Walker, 1856)

= *severini* (Goetghebuer, 1923)

*Glyptotendipes scirpi* (Kieffer, 1915)

= *fodiens* (Kieffer, 1924)

= *mancunianus* (Edwards, 1929 )

*Glyptotendipes viridis* (Macquart, 1834)

**sg. *Glyptotendipes*** Kieffer, 1913

*Glyptotendipes barbipes* (Staeger, 1839)

*Glyptotendipes cauliginellus* (Kieffer, 1913)

= *gripekoveni* (Kieffer, 1913)

*Glyptotendipes glaucus* (Meigen, 1818)

*Glyptotendipes pallens* (Meigen, 1804)

*Glyptotendipes paripes* (Edwards, 1929)

**sg. *Heynotendipes*** Spies & Sæther, 2004

*Glyptotendipes signatus* (Kieffer, 1909)

***HARNISCHIA*** Kieffer, 1921

*Harnischia curtilamellata* (Malloch, 1915)

*Harnischia fuscimana* Kieffer, 1921

***KIEFFERULUS*** Goetghebuer, 1922

*Kiefferulus tendipediformis* (Goetghebuer, 1921)

***KLOOSIA*** Kruseman, 1933

*Kloosia pusilla* (Linnaeus, 1767)

***LAUTERBORNIELLA*** Thienemann & Bause, 1913

*Lauterborniella agrayloides* (Kieffer, 1911)

***LIPINIELLA*** Shilova, 1961

? *Lipiniella araenicola* Shilova, 1961 (larval ident.)

*Lipiniella prima* Shilova, Kerkis & Kiknadze, 1993

***MICROCHIRONOMUS*** Kieffer, 1918

*Microchironomus tener* (Kieffer, 1918)

***MICROTENDIPES*** Kieffer, 1915

*Microtendipes brevitarsis* Brundin, 1947

*Microtendipes chloris* (Meigen, 1818)

*Microtendipes confinis* (Meigen, 1830)

*Microtendipes nigellus* Hirvenoja, 1963

*Microtendipes pedellus* (De Geer, 1776)

*Microtendipes rydalensis* (Edwards, 1929)

***NILOTHAUMA*** Kieffer, 1921

*Nilothauma brayi* (Goetghebuer, 1921)

***OMISUS*** Townes, 1945

*Omisus caledonicus* (Edwards, 1932)

***PAGASTIELLA*** Brundin, 1949

*Pagastiella orophila* (Edwards, 1929)

***PARACHIRONOMUS*** Lenz, 1921

*Parachironomus biannulatus* (Staeger, 1839)

*Parachironomus digitalis* (Edwards, 1929)

*Parachironomus frequens* (Johannsen, 1905)

*Parachironomus gracilior* (Kieffer, 1918)

= *arcuatus* (Goetghebuer, 1919)

*Parachironomus monochromus* (van der Wulp, 1874)

*Parachironomus paradigitalis* Brundin, 1949

*Parachironomus parilis* (Walker, 1856)

Parachironomus cf. pseudovarus Zorina, 2003

*Parachironomus siljanensis* Brundin, 1949

*Parachironomus subalpinus* (Goetghebuer, 1932)

*Parachironomus tenuicaudatus* (Malloch, 1915)

*Parachironomus varus* (Goetghebuer, 1921)

*Parachironomus vitiosus* (Goetghebuer, 1921)

*Parachironomus* sp. 1 “Iijoki”

***PARACLADOPELMA*** Harnisch, 1923

*Paracladopelma camptolabis* (Kieffer, 1913)

*Paracladopelma galaptera* (Townes, 1945)

*Paracladopelma laminatum* (Kieffer, 1921)

*Paracladopelma nereis* (Townes, 1945)

*Paracladopelma nigritulum* (Goetghebuer, 1942)

*Paracladopelma undine* (Townes, 1945)

***PARALAUTERBORNIELLA*** Lenz, 1941

*Paralauterborniella nigrohalteralis* (Malloch, 1915)

***PARATENDIPES*** Kieffer, 1911

*Paratendipes albimanus* (Meigen, 1818)

*Paratendipes subaequalis* (Malloch, 1915)

*Paratendipes* conn. no. 3 Lipina, 1926 (larval type)

***PHAENOPSECTRA*** Kieffer, 1921

*Phaenopsectra flavipes* (Meigen, 1818)

*Phaenopsectra punctipes* (Wiedemann, 1817)

***POLYPEDILUM*** Kieffer, 1912

**sg. *Pentapedilum*** Kieffer, 1913

*Polypedilum sordens* (van der Wulp, 1874)

*Polypedilum tritum* (Walker, 1856)

? = *uncinatum* (Goetghebuer, 1921)

**sg. *Polypedilum*** Kieffer, 1912

*Polypedilum acutum* Kieffer, 1915

*Polypedilum albicorne* (Meigen, 1838)

*Polypedilum amoenum* Goetghebuer, 1930

*Polypedilum arundineti* (Goetghebuer, 1921)

*Polypedilum laetum* (Meigen, 1818)

*Polypedilum nubeculosum* (Meigen, 1804)

*Polypedilum pedestre* (Meigen, 1830)

*Polypedilum trigonus* Townes, 1945

**sg. *Tripodura*** Townes, 1945

*Polypedilum aegyptium* Kieffer, 1925

*Polypedilum bicrenatum* Kieffer, 1921

*Polypedilum pullum* (Zetterstedt, 1838)

*Polypedilum quadriguttatum* Kieffer, 1921

*Polypedilum scalaenum* (Schrank, 1803)

*Polypedilum tetracrenatum* Hirvenoja, 1962

**sg. *Uresipedilum*** Sasa & Kikuchi, 1995

*Polypedilum convictum* (Walker, 1856)

*Polypedilum cultellatum* Goetghebuer, 1931

***ROBACKIA*** Sæther, 1977

*Robackia pilicauda* Sæther, 1977

? *Robackia demeijerei* (Kruseman, 1933) (larval ident.)

***SAETHERIA*** Jackson, 1977

*Saetheria reissi* Jackson, 1977

***SERGENTIA*** Kieffer, 1922

*Sergentia baueri* Wülker, Kiknadze, Kerkis & Nevers, 1999

*Sergentia coracina* (Zetterstedt, 1850)

*Sergentia prima* Proviz & Proviz, 1997

***STENOCHIRONOMUS*** Kieffer, 1919

**sg. *Petalopholeus*** Borkent, 1984

*Stenochironomus fascipennis* (Zetterstedt, 1838)

**sg. *Stenochironomus*** Kieffer, 1919

*Stenochironomus gibbus* (Fabricius, 1794)

*Stenochironomus hibernicus* (Edwards, 1929)

***STICTOCHIRONOMUS*** Kieffer, 1919

*Stictochironomus crassiforceps* (Kieffer, 1921)

*Stictochironomus maculipennis* (Meigen, 1818)

*Stictochironomus pictulus* (Meigen, 1830)

*Stictochironomus rosenschoeldi* (Zetterstedt, 1838)

*Stictochironomus sticticus* (Fabricius, 1781)

***SYNENDOTENDIPES*** Grodhaus, 1987

*Synendotendipes dispar* (Meigen, 1830)

*Synendotendipes impar* (Walker, 1856)

*Synendotendipes lepidus* (Meigen, 1830)

***TRIBELOS*** Townes, 1945

Tribelos cf. donatoris (Shilova, 1974 )

?= *subatrum* Grodhaus, 1987

*Tribelos intextum* (Walker, 1856)

***XENOCHIRONOMUS*** Kieffer, 1921

*Xenochironomus xenolabis* (Kieffer, 1916)

***ZAVRELIELLA*** Kieffer, 1920

*Zavreliella marmorata* (van der Wulp, 1859)

tribe Pseudochironomini Sæther, 1977

***PSEUDOCHIRONOMUS*** Malloch, 1915

*Pseudochironomus prasinatus* (Staeger, 1839)

tribe Tanytarsini Zavřel, 1917

***CLADOTANYTARSUS*** Kieffer, 1921

**sg. *Cladotanytarsus*** Kieffer, 1921

*Cladotanytarsus atridorsum* Kieffer, 1924

*Cladotanytarsus cyrylae* Giłka, 2001

*Cladotanytarsus difficilis* Brundin, 1947

*Cladotanytarsus gedanicus* Giłka, 2001

*Cladotanytarsus iucundus* Hirvenoja, 1962

*Cladotanytarsus lepidocalcar* Krüger, 1938

*Cladotanytarsus mancus* (Walker, 1856)

*Cladotanytarsus matthei* Giłka, 2001

*Cladotanytarsus molestus* Hirvenoja, 1962

*Cladotanytarsus nigrovittatus* (Goetghebuer, 1922)

*Cladotanytarsus pallidus* Kieffer, 1922

*Cladotanytarsus teres* Hirvenoja, 1962

*Cladotanytarsus vanderwulpi* (Edwards, 1929)

**sg. *Lenziella*** Kieffer, 1922

*Cladotanytarsus amandus* Hirvenoja, 1962

*Cladotanytarsus bicornutus* Kieffer, 1922

= *wexionensis* Brundin, 1947

***CONSTEMPELLINA*** Brundin, 1947

*Constempellina brevicosta* (Edwards, 1937)

***CORYNOCERA*** Zetterstedt, 1837

*Corynocera ambigua* Zetterstedt, 1837

*Corynocera oliveri* Lindeberg, 1970

***MICROPSECTRA*** Kieffer, 1908

= ***Lauterbornia*** Kieffer, 1911

= ***Lundstroemia*** Kieffer, 1921

= ***Parapsectra*** Reiss, 1969

*Micropsectra appendica* Stur & Ekrem, 2006

*Micropsectra apposita* (Walker, 1856)

*Micropsectra atrofasciata* (Kieffer, 1911)

*Micropsectra attenuata* Reiss, 1969

*Micropsectra calcifontis* Stur & Ekrem, 2006

*Micropsectra chionophila* (Edwards, 1933)

*Micropsectra insignilobus* Kieffer, 1924

*Micropsectra junci* (Meigen, 1818)

*Micropsectra klinki* Stur & Ekrem, 2006

*Micropsectra lacustris* Säwedal, 1975

*Micropsectra lindebergi* Säwedal, 1976

*Micropsectra logani* (Johannsen, 1928)

= *groenlandica* Soegaard Andersen, 1937

*Micropsectra malla* Giłka & Paasivirta, 2008

*Micropsectra nana* (Meigen, 1818)

*Micropsectra notescens* (Walker, 1856)

*Micropsectra pallidula* (Meigen, 1830)

= *bidentata* auct.

*Micropsectra radialis* Goetghebuer, 1939

= *coracina* (Kieffer, 1911)

*Micropsectra recurvata* Goetghebuer, 1928

*Micropsectra rilensis* Giłka, 2001

*Micropsectra roseiventris* (Kieffer, 1909)

= *fusca* auct. nec (Meigen, 1804)

*Micropsectra schrankelae* Stur & Ekrem, 2006

*Micropsectra sofiae* Stur & Ekrem, 2006

*Micropsectra styriaca* Reiss, 1969

***NEOZAVRELIA*** Goetghebuer & Thienemann, 1941

*Neozavrelia cuneipennis* (Edwards, 1929)

***PARATANYTARSUS*** Thienemann & Bause, 1913

*Paratanytarsus abiskoensis* Reiss & Säwedal, 1981

*Paratanytarsus austriacus* (Kieffer, 1924)

*Paratanytarsus bituberculatus* (Edwards, 1929)

*Paratanytarsus brevicalcar* (Kieffer, 1909)

= *intricatus* (Goetghebuer, 1921)

*Paratanytarsus dimorphis* Reiss, 1965

*Paratanytarsus dissimilis* (Johannsen, 1905)

= *confusus* Palmén, 1960

*Paratanytarsus grimmii* (Schneider, 1885)

= *boiemicus* Bause, 1913

*Paratanytarsus hyperboreus* Brundin, 1949

*Paratanytarsus inopertus* (Walker, 1856)

*Paratanytarsus laccophilus* (Edwards, 1929)

*Paratanytarsus laetipes* (Zetterstedt, 1850)

*Paratanytarsus lauterborni* (Kieffer, 1909)

*Paratanytarsus natvigi* (Goetghebuer, 1933)

*Paratanytarsus paralaccophilus* Giłka & Paasivirta, 2008

*Paratanytarsus penicillatus* (Goetghebuer, 1928)

*Paratanytarsus tenellulus* (Goetghebuer, 1921)

*Paratanytarsus tenuis* (Meigen, 1830)

***RHEOTANYTARSUS*** Thienemann & Bause, 1913

*Rheotanytarsus curtistylus* (Goetghebuer, 1921)

*Rheotanytarsus muscicola* Thienemann, 1929

*Rheotanytarsus pellucidus* (Walker, 1848)

= *distinctissimus* (Brundin, 1947)

*Rheotanytarsus pentapoda* (Kieffer, 1909)

*Rheotanytarsus photophilus* (Goetghebuer, 1921)

*Rheotanytarsus ringei* Lehmann, 1970

***STEMPELLINA*** Thienemann & Bause, 1913

*Stempellina almi* Brundin, 1947

*Stempellina bausei* (Kieffer, 1911)

*Stempellina subglabripennis* (Brundin, 1947)

*Stempellina tervolae* Giłka, 2005

***STEMPELLINELLA*** Brundin, 1947

*Stempellinella brevis* (Edwards, 1929)

*Stempellinella edwardsi* Spies & Sæther, 2004

= *minor* (Edwards, 1929) preocc.

*Stempellinella flavidula* (Edwards, 1929)

*Stempellinella saltuum* (Goetghebuer, 1921)

***TANYTARSUS*** van der Wulp, 1874

*Tanytarsus aberrans* Lindeberg, 1970

*Tanytarsus aculeatus* Brundin, 1949

*Tanytarsus anderseni* Reiss & Fittkau, 1971

*Tanytarsus bathophilus* Kieffer, 1911

= *tripunctatus* Reiss, 1968

*Tanytarsus brundini* Lindeberg, 1963

*Tanytarsus buchonius* Reiss & Fittkau, 1971

*Tanytarsus chinyensis* Goetghebuer, 1934

*Tanytarsus curticornis* Kieffer, 1911

*Tanytarsus debilis* (Meigen, 1830)

*Tanytarsus desertor* Giłka & Paasivirta, 2007

*Tanytarsus dibranchius* Kieffer, 1926

= *separabilis* Brundin, 1947

*Tanytarsus dispar* Lindeberg, 1967

*Tanytarsus eminulus* (Walker, 1856)

*Tanytarsus excavatus* Edwards, 1929

*Tanytarsus fennicus* Lindeberg, 1970

*Tanytarsus gibbosiceps* Kieffer, 1922

*Tanytarsus glabrescens* Edwards, 1929

*Tanytarsus gracilentus* (Holmgren, 1883)

*Tanytarsus gregarius* Kieffer, 1909

*Tanytarsus heusdensis* Goetghebuer, 1923

*Tanytarsus inaequalis* Goetghebuer, 1921

*Tanytarsus innarensis* Brundin, 1947

*Tanytarsus lactescens* Edwards, 1929

*Tanytarsus lapponicus* Lindeberg, 1970

*Tanytarsus latiforceps* Edwards, 1941

*Tanytarsus lestagei* Goetghebuer, 1922

= *decipiens* Lindeberg, 1967

= *palmeni* Lindeberg, 1967

*Tanytarsus longitarsis* Kieffer, 1911

*Tanytarsus lugens* (Kieffer, 1916)

*Tanytarsus mancospinosus* Ekrem & Reiss, 1999

*Tanytarsus medius* Reiss & Fittkau, 1971

*Tanytarsus mendax* Kieffer, 1925

= *holochlorus* Edwards, 1929

*Tanytarsus miriforceps* (Kieffer, 1921)

*Tanytarsus multipunctatus* Brundin, 1947

*Tanytarsus nemorosus* Edwards, 1929

*Tanytarsus niger* Soegaard Andersen, 1937

*Tanytarsus norvegicus* (Kieffer, 1924)

*Tanytarsus occultus* Brundin, 1949

*Tanytarsus palettaris* Verneaux, 1969

*Tanytarsus pallidicornis* (Walker, 1856)

*Tanytarsus paraniger* Giłka & Paasivirta, 2008

*Tanytarsus quadridentatus* Brundin, 1947

*Tanytarsus recurvatus* Brundin, 1947

*Tanytarsus salmelai* Giłka & Paasivirta, 2009

*Tanytarsus signatus* (van der Wulp, 1859)

*Tanytarsus smolandicus* Brundin, 1947

*Tanytarsus striatulus* Lindeberg, 1976

*Tanytarsus sylvaticus* (van der Wulp, 1859)

= *aptus* Hirvenoja, 1963

*Tanytarsus telmaticus* Lindeberg, 1959

= *simulans* Lindeberg, 1967

= *socialis* Lindeberg, 1967

*Tanytarsus trux* Giłka & Paasivirta, 2007

*Tanytarsus usmaensis* Pagast, 1931

*Tanytarsus verralli* Goetghebuer, 1928

*Tanytarsus volgensis* Miseiko, 1967

= *fimbriatus* Reiss & Fittkau, 1971

***VIRGATANYTARSUS*** Pinder, 1982

*Virgatanytarsus arduennensis* (Goetghebuer, 1922)

***ZAVRELIA*** Kieffer, Thienemann & Bause, 1913

*Zavrelia pentatoma* Kieffer & Bause, 1913

= *atrofasciata* Kieffer, 1921

## Excluded species

The numerous species that were reported by Hackman (1980), but are excluded from the current check-list, are not listed here. Reasons for deletions of those species are various, mainly due to misidentifications and nomenclatural changes.

## Notes

***Chironomus pallidivittatus* Edwards, 1929**. This name is preoccupied by *Chironomus pallidivittatus* Malloch, 1915. The European species needs a new name.
